# Association between the Delta Estimated Glomerular Filtration Rate and the Prevalence of Monoclonal Gammopathy of Undetermined Significance in Korean Males

**DOI:** 10.1155/2014/356080

**Published:** 2014-05-08

**Authors:** Tae-Dong Jeong, Woochang Lee, Sail Chun, Won-Ki Min

**Affiliations:** Department of Laboratory Medicine, Asan Medical Center, University of Ulsan College of Medicine, 88 Olympic-ro 43-gil, Songpa-gu, Seoul 138-736, Republic of Korea

## Abstract

*Background*. We investigated the association between the reduction in the estimated glomerular filtration rate (eGFR) and the prevalence of monoclonal gammopathy of undetermined significance (MGUS) in Korean males. *Methods*. We enrolled 723 healthy Korean males. Serum creatinine concentration, serum electrophoresis, serum immunofixation, and the serum free light chain assay were performed. We calculated delta eGFR per year (ΔeGFR/yr). The prevalence of MGUS was compared based on the ΔeGFR/yr and age group. *Results*. Thirteen (1.8%) of 723 participants exhibited the monoclonal band on serum immunofixation. Prevalence of MGUS by age group was 0.00% (0/172 for 40 years), 1.63% (6/367 for 60 years), and 3.80% (7/184 for >60 years). The median decrease in ΔeGFR/yr was 5.3%. The prevalence of MGUS in participants in their 50s with >5.3% decline in ΔeGFR/yr was significantly higher than those with <5.3% decrease in ΔeGFR/yr (3.16% versus 0.00%; *P* = 0.049). The prevalence of MGUS in participants in their 50s with >5.3% decrease in ΔeGFR/yr was similar to that of healthy males in their 60s. *Conclusion*. Using the rate of reduction in ΔeGFR/yr in healthy Korean males who had their serum creatinine level checked regularly may increase the MGUS detection rate in clinical practice.

## 1. Introduction

Monoclonal gammopathy of undetermined significance (MGUS) is the most common plasma cell disorder, and its prevalence increases with age [1–4]. The prevalence of MGUS is also affected by race and sex, but MGUS generally develops in older people, African-Americans, and males with a slight predominance [1–5]. In previous studies, the prevalence of MGUS was 4.9% in Caucasian males > 60 years old [2] and 3.8% in Korean males > 65 years old [3].

Several studies of the prevalence of MGUS stratified by age category in each race have been conducted. However, to the best of our knowledge, none has reported the correlation between the degree of estimated glomerular filtration rate (eGFR) reduction and the prevalence of MGUS. And the association between measurement of free kappa and lambda light chains by the serum FLC assay and the prevalence of MGUS has not been well documented either. Thus, in this study we investigated the correlation between the degree of egfr reduction and MGUS prevalence among Korean healthy males.

## 2. Materials and Methods

### 2.1. Subjects

The study included Korean males over 40 years old who visited the hospital for regular health checkups between June 2011 and March 2012. A total of 723 males had regular checkups during the period. According to the review of the electronic medical records, all of the study subjects have not been diagnosed with MGUS previously. This study was approved by the Institutional Review Board of ASAN Medical Center (approval no. 2012-0065).

### 2.2. Specimen and Data Collection

After general blood chemistry, the remaining 1 mL of serum was collected in two microtubes and stored at −70°C. One of the two microtubes was used for serum protein electrophoresis (sPEP) and serum immunofixation electrophoresis (sIFE), while the other was used for the serum free light chain (sFLC) assay. Electronic medical records were used to collect participant data, such as age, date of current health checkup, date of previous health checkup, and current and previous serum creatinine levels.

### 2.3. Laboratory Tests

Serum creatinine levels of the participants during the study period were measured using a Toshiba 200-FR Neo (Toshiba Medical Systems Co., Tokyo, Japan) instrument with the IDMS-traceable calibrator (c.f.a.s calibrator, Roche Diagnostics, Indianapolis, IN, USA). Previous serum creatinine levels were measured using the Toshiba 200-FR Neo with the same IDMS-traceable calibrator. The Hydrasys 2 (Sebia, Evry, France) instrument was used to perform both sPEP and sIFE based on the manufacturer's instructions. The sIFE was performed to detect kappa and lambda free light chains in sera. The sFLC assay was analyzed using SPAplus (Binding Site, Birmingham, UK) instrument with Freelite (Binding Site) reagents. Both kappa and lambda free light chain concentrations were measured quantitatively to calculate the kappa/lambda FLC ratio. The reference range for the sFLC ratio was 0.26–1.65 [6].

### 2.4. Calculating eGFR and ΔeGFR

All participants were Korean males. Their current and previous eGFR values were calculated using the four-variable MDRD study equation (eGFR = 175 × standardized serum creatinine^−1.154^ × age^−0.203^). The delta eGFR (ΔeGFR, %) was calculated as the difference between current eGFR and previous eGFR [100 × (current eGFR-previous eGFR)/previous eGFR], whereas the annual rate of decline in eGFR [ΔeGFR/yr (%)] was determined as the difference between the follow-up and baseline eGFR values, with this value divided by the time interval [100 × (current eGFR-previous eGFR)/previous eGFR] × (365/Δdays).

### 2.5. Definition of MGUS

MGUS is defined as serum monoclonal protein <3 g/dL, clonal bone marrow clonal plasma cells <10%, and absence of end organ damage such as hypercalcemia, renal insufficiency, anemia, and bone lesions (CRAB) that can be attributed to the plasma cell proliferation disorder [7]. In this study,MGUS was defined as an absence of CRAB symptoms and presence of monoclonal protein confirmed by sIFE. The CRAB symptoms were identified through review of the electronic medical records including laboratory findings.

### 2.6. Statistical Analyses

The participants were grouped by age (40, 50, and >60 years) and ΔeGFR/yr (classified into two groups by median ΔeGFR/yr). The prevalence of MGUS was calculated for each group and the differences were analyzed by the chi-square test or Fisher's exact test. SPSS version 19.0 (SPSS, Inc., Chicago, IL, USA) was used for statistical analyses. A *P* value <0.05 was considered to indicate significance.

## 3. Results

### 3.1. Baseline Characteristics

The mean age of the participants was 55.4 ± 7.4 years (range, 41–90 years) and the average health checkup interval was 401.3 ± 99.4 days (range, 202–768 days). The current serum creatinine level increased by an average of 0.051 ± 0.089 mg/dL from the previous serum creatinine level. eGFR decreased by an annual average of 5.3 ± 8.5%, and 363 participants had ≥5.3% reduction. Median values of serum kappa FLC concentration, serum lambda FLC concentration, and the serum kappa/lambda FLC ratio were 11.10 mg/L, 11.84 mg/L, and 0.92, respectively. The baseline demographic chracteristics of the study population stratified by age are summarized in Table 1.

### 3.2. sPEP, sIFE, and sFLC Assays

The monoclonal protein was detected in 9 participants by sPEP, 13 by sIFE, and 21 by the sFLC ratio, respectively. Four participants had the monoclonal protein in all three tests. The monoclonal protein was detected in 4 participants by only sIFE and in 17 by only the sFLC assay.

### 3.3. Prevalence of MGUS

Thirteen (1.80%) of the seven hundred and twenty-three participants had the monoclonal band on sIFE. The prevalence of MGUS by age group was 0.00% (0/172, 40 years of age), 1.63% (6/367, 50 years of age), and 3.80% (7/184, ≥60 years of age) (Table 2).

**Figure 1:**
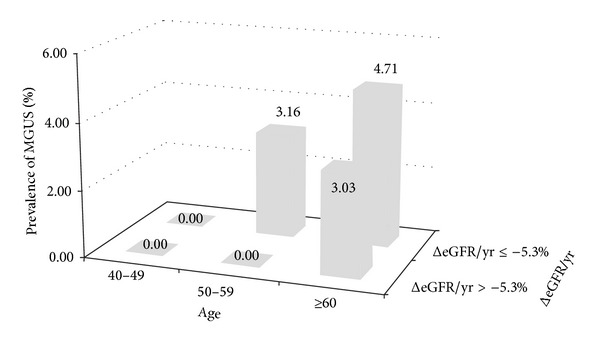
Prevalence of monoclonal gammopathy of undetermined significance in Korean males based on delta estimated glomerular filtration rate and age.

The MGUS prevalence in the group with ≥5.3% decline in ΔeGFR/yr was 2.75% (10/363), almost threefold that in the other groups (0.83%, 3/360); however, the difference was not significant (*P* = 0.089). In an age-stratified analyses, the prevalence of MGUS in 50s with ≥5.3% decline in ΔeGFR/yr group was significantly higher than those with <5.3% decline in ΔeGFR/yr group (3.16% versus 0.00%; *P* = 0.049) (Figure 1). In the 60 years of age or older group, however, the prevalence of MGUS showed no significant difference based on ΔeGFR/yr (4.71% versus 3.03%; *P* = 0.438) (Figure 1).

The MGUS prevalence in participants with kappa or lambda FLC concentrations higher than the reference value on sFLC was 10.91% (6/55), significantly higher than the 1.05% (7/668) in those with a normal serum kappa and lambda FLC concentration.

## 4. Discussion

We investigated the association between ΔeGFR/yr and prevalence of MGUS in healthy Korean males aged over 40 years. Our results showed that MGUS prevalence values in Korean men in their 40 (0.00%), 50 (1.63%), and >60 years of age (3.80%) were similar to MGUS values for Japanese males in their 40 (1.2%), 50 (2.7%), and >60 years of age (4.3%) and Chinese men 50–65 years (1.2%) [8, 9]. To date, the MGUS prevalence in Koreans has been determined only in elderly people >65 years [3]. In the present study, the prevalence of MGUS in participants >60 years was 3.80% which was similar to those obtained from elderly Korean male aged over 65 years (3.8%) [3].

By definition of MGUS, there is no renal insufficiency. However, excess serum free light chains and intact immunoglobulins that exceed the metabolic ability of kidney function in patients with MGUS can accumulate in the kidney, resulting in subclinical renal insufficiency such as a decline in GFR [10, 11]. On the basis of above consideration, we had assumed that the prevalence of MGUS may be higher in patients with markedly reduction of GFR. And the GFR decline may occur within reference range of serum creatinine. In our study, although the prevalence of MGUS in all participants was not significantly different based on the ΔeGFR, a statistically significant difference was found in the group in their 50s. The difference between the 50s group and those >60 can be partially explained that MGUS is not always a premalignant lesion. Our study indicated that there was apparent association between the MGUS and ΔeGFR; however, a large-scale cohort study is needed to reach the statistical significance.

According to the current MGUS management guideline, MGUS does not require aggressive treatment [12]. However, MGUS is an obvious premalignant lesion that requires careful observation because it progresses to multiple myeloma or other plasma cell-related disorders in some patients [12–15]. In this study, the MGUS prevalence in the group with ≥5.3% decline in ΔeGFR/yr was almost threefold that in the other groups. The prevalence of MGUS in participants in their 50s with a high rate of decrease in ΔeGFR/yr was 3.16%, which was similar to that in the group in their 60s. As 10–20% of those with MGUS in this group are likely to develop multiple myeloma or diverse malignant plasma cell disorders when they reach 60–70 years of age, early detection of MGUS in 50s should be considered.

Measurement of serum creatinine level is included in the National Health Screening Program organized by the National Health Insurance Service for Koreans who are required to undergo a regular health checkup every year or 2 years. And the ΔeGFR can be calculated from those data. We thought that addition of screening for monoclonal protein in those whose ΔeGFR/yr shows a marked reduction may increase the MGUS diagnostic rate.

In this study, we observed 9 samples of false-negative results for sFLC assay based on the sIFE findings. We thought that possible sources of false-negative results of sFLC assay were antigen excess [16], FLC polymerization [17], and polyclonal FLC elevation [18]. On the other hand, sFLC assay demonstrated false-positive results for 17 samples based on the sIFE findings. Seventeen participants were normal on the sIFE and sPEP tests but had an abnormal sFLC ratio. Among them, 15 participants showed kappa clonality which means that their sFLC ratio showed more than 1.65 and the remaining 2 participants demonstrated lambda clonality which means that their sFLC ratio is less than 0.26 (data not shown). It has been well documented that sFLC assay could detect monoclonal protein more sensitive than sPEP and sIFE assay [6, 19]. So it is not clear whether 17 samples are true false-positive results. These 17 participants may have monoclonal protein in their sera. So we thought that they need to be monitored to identify monoclonal protein.

In conclusion, the prevalence of MGUS in healthy Korean males in their 50s with a decrease in ΔeGFR/yr of ≥5.3% was significantly higher than those with a decrease in ΔeGFR/yr of <5.3%. Additionally, the prevalence of MGUS was similar to that of healthy Korean males in their 60s. Using ΔeGFR/yr in healthy Korean males whose serum creatinine level is checked regularly may facilitate early detection of MGUS.

## Figures and Tables

**Table 1:** Baseline demographic chracteristics of the study population stratified by age.

Variable	40–49 yrs	50–59 yrs	≥60 yrs	All
*N*	172	367	184	723
Age (yrs)	47.0 ± 1.7	54.3 ± 2.9	65.4 ± 5.2	55.4 ± 7.4
Interval of health checkup (days)	395.6 ± 97.3	394.5 ± 91.7	420.2 ± 113.4	401.3 ± 99.4
Current sCr (mg/dL)	0.989 ± 0.123	0.996 ± 0.155	1.015 ± 0.155	0.999 ± 0.148
Previous sCr (mg/dL)	0.943 ± 0.113	0.941 ± 0.117	0.968 ± 0.129	0.949 ± 0.120
Current eGFR (mL/min/1.73 m^2^)	82.6 ± 11.5	80.2 ± 12.6	75.6 ± 12.8	79.6 ± 12.7
Previous GFR (mL/min/1.73 m^2^)	87.7 ± 12.6	85.3 ± 11.9	79.8 ± 12.7	84.5 ± 12.6
ΔeGFR/yr (%)	−5.1 ± 9.0	−5.6 ± 8.5	−4.7 ± 8.0	−5.3 ± 8.5
*κ* FLC (mg/L)	9.77 (2.38–25.91)	10.76 (2.17–292.53)	11.54 (2.17–1,265.95)	11.10 (2.17–1,265.95)
*λ* FLC (mg/L)	11.13 (6.38–30.31)	11.71 (2.51–34.71)	12.06 (2.51–34.71)	11.84 (2.51–34.71)
*κ*/*λ* ratio	0.84 (0.19–1.99)	0.91 (0.33–44.26)	0.94 (0.33–156.68)	0.92 (0.19–156.68)

Data expressed as means ± standard deviation or medians (range).

Abbreviations: CKD-EPI: Chronic Kidney Disease Epidemiology Collaboration; eGFR: estimated glomerular filtration rate; FLC: free light chain; MDRD: Modification of Diet in Renal Disease; sCr: serum creatinine.

**Table 2:** Prevalence of monoclonal gammopathy of undetermined significance (MGUS) in Korean males.

Age (yrs)	Total number of patients	Number of patients with MGUS	Prevalence of MGUS
40–49	172	0	0.00%
50–59	367	6	1.63%
≥60	184	7	3.80%

All	723	13	1.80%
